# The acute effects of plyometric and sled towing stimuli with and without caffeine ingestion on vertical jump performance in professional soccer players

**DOI:** 10.1186/s12970-018-0258-3

**Published:** 2018-10-22

**Authors:** Mauro A. Guerra Jr, Leonardo C. Caldas, Helder L. De Souza, Kaio F. Vitzel, Jason M. Cholewa, Michael J. Duncan, Lucas Guimarães-Ferreira

**Affiliations:** 1 Muscle Physiology and Human Performance Research Group, Center of Physical Education and Sports, Federal University of Espirito Santo, Av. Fernando Ferrari, 514, Campus Universitário, Goiabeiras, Vitória, ES 29075-810 Brazil; 2 Massey Institute of Food Science and Technology, College of Health, Massey University, Palmerston North, New Zealand; 3 Department of Kinesiology, Coastal Carolina University, Conway, SC USA; 4 Faculty of Health and Life Sciences, Coventry University, Coventry, UK

**Keywords:** Caffeine, Post-activation potentiation, Vertical jump, Sports performance

## Abstract

**Background:**

Post-activation potentiation (PAP) is the phenomenon by which muscular performance is enhanced in response to a conditioning stimulus. PAP has typically been evidenced via improved counter movement jump (CMJ) performance. This study examined the effects of PAP, with and without prior caffeine ingestion, on CMJ performance.

**Methods:**

Twelve male professional soccer players (23 ± 5 years) performed two trials of plyometric exercises and sled towing 60 min after placebo or caffeine ingestion (5 mg.kg^− 1^) in a randomized, counterbalanced and double-blinded design. CMJ performance was assessed at baseline and 1, 3 and 5 min after the conditioning stimulus (T1, T3 and T5, respectively).

**Results:**

Two way ANOVA main effects indicated a significant difference in jump height after the PAP protocol (F[3, 11] = 14.99, *P* < 0.001, partial η2 = 0.577). Analysis also indicated a significant difference in CMJ performance across conditions, with caffeine eliciting a greater response (F[1, 11] = 10.12, *P* = 0.009, partial η2 = 0.479). CMJ height was increased at T1, T3 and T5 in caffeine condition (5.07%, 5.75% and 5.40%, respectively; *P* < 0.01) compared to baseline. In the placebo condition, jump performance was increased at T3 (4.94%; *P* < 0.01) only. Jump height was higher in caffeine condition on T1, T3 and T5 (*P* < 0.05) but not on baseline (*P* > 0.05) compared to placebo.

**Conclusions:**

The results of this study suggest that acute plyometric and sled towing stimuli enhances jump performance and that this potentiation is augmented by caffeine ingestion in male soccer players.

## Background

Post-activation potentiation (PAP), also known as activity-dependent potentiation, is the phenomenon by which muscular performance is enhanced in response to a conditioning stimulus [29]. It has been applied in sports where enhanced force production, speed, and power are required [18]. A body of evidence supports that previous, heavy, near maximum strength exercises improve performance in a subsequent exercise bout. For example, Duncan et al. [9] demonstrated that three repetitions of back squat with a load of 90% of 1 repetition maximum, 4 min prior to repeated sprints increased performance (speed and fatigue rate) in professional union rugby players. Other studies have used submaximal dynamic contractions to elicit a PAP response, whilst others have used maximal isometric contractions with equivocal results (reviewed by [18]). The large inter-individual response variation should also be considered and may explain the contradictory results among studies. For example, Till and Cooke [34] reported a variation of individual responses to sprint and vertical jump performance of − 7.1 to + 8.2% in male soccer players after a PAP protocol.

Few studies have used plyometric exercise as a potentiating stimulus. Recently, Tobin and Delahunt [36] demonstrated that plyometric exercises were able to elicit a potentiating response in professional rugby players. The intervention consisted of 2 sets of 10 ankle hops, 3 sets of 5 hurdle hops, and 5 drop jumps from a height of 50 cm, resulting in a total of 40 jumps. Countermovement jump (CMJ) height and peak force were significantly increased after 1, 3 and 5 min, compared to baseline. Similarly, De Villareal et al. [6] reported that a volleyball-specific warm-up consisting of 4 different plyometric exercises (5 × two-foot ankle hops; 5 × split squat jump; 5 × standing jump and reach and 10 × rim jump) improved CMJ height by 3.08 cm 5 min after the stimulus. In contrast, two other studies reported no effect of a plyometric stimulus on subsequent jump performance. The volume and/or intensity of the plyometric exercises used, or the rest time after the stimulus may explain the discrepancy observed among studies. For example, Till and Cooke [34] used 5 double-leg tuck jumps, which is considered a low volume compared to other studies. In turn, Esformes et al. [10] used 1 set of 6 ft contacts for each exercise used (alternate speed bounds, single leg speed hops and vertical bounds). While CMJ performance was evaluated 7, 8, and 9 min after the stimulus at the former, a 10 min rest period was used at the latter study. When compared to the control trial, no improvement in jump performance was observed in both studies.

While PAP alone offers an attractive and practical means for conditioning coaches to elicit and increase in muscle performance there are other means by which coaches can enhance muscle performance that may be synergistic when combined with PAP. In particular, caffeine (1,3,7 trimethylxanthine) ingestion has been shown to enhance explosive exercise performance [16]. For example, Lara et al. [20] reported that ingestion of a drink containing 3 mg.kg-1 of caffeine resulted in 3.0% improvement of CMJ performance in female soccer players. Similarly, Abian et al. [1] demonstrated that ingestion of the same dose of caffeine improved CMJ performance in 4,7% in male elite badminton players. Similar results were reported by others [2, 7, 8, 11, 13, 27, 32]. Given the widespread use of both caffeine and PAP as a means to enhance short term exercise performance, it would be of interest to investigate whether the PAP effect might be augmented in the presence of caffeine ingestion.

To the best of our knowledge, the combined effect of PAP and caffeine ingestion on CMJ performance has not been investigated. Therefore, the aim of this study was to examine the effects of a combined plyometric and sled towing stimulus (the conditioning activity) on PAP response with and without caffeine ingestion on CMJ jump performance in professional soccer players. It was hypothesized that (a) both the conditioning would induce an enhancement of CMJ performance; and (b) the PAP protocol would result in a greater impact on performance when combined with previous caffeine ingestion.

## Methods

### Participants

Twenty-eight professional soccer athletes were recruited from a soccer club. Habitual caffeine consumption was determined through a caffeine consumption questionnaire (based on data presented by [24]) and to control individual differences in reactivity to caffeine from habituation, only participants with daily caffeine intake of less than 250 mg.d^−1^were included. Fourteen players matched this criterion but two dropped out of the study due to injury, resulting in the inclusion of 12 players in this study. The characteristics of the participants are presented in Table 1. All subjects were accustomed to the plyometric and sled towing exercises required for the study. The tests were conducted during the regular 2015 season, and all players were living at the club facility during weekdays and were subjected to the same exercise and diet regimen during the data collection period. The institution’s Human Research Ethics Committee approved the procedures used in this study and all the athletes gave informed consent to take part, in accordance with the 1964 Declaration of Helsinki.

**Table 1:** Subjects’ characteristics (Mean ± SD)

	Mean ± SD
Age (years)	23.83 ± 5.06
Body weight (Kg)	79.5 ± 9.13
Body fat (%)	12.66 ± 2.76
Caffeine intake (mg.day^− 1^)	110.83 ± 60.83

### Measures

This study used a within-subjects, repeated-measures, and double-blinded controlled design. The investigation employed 12 male professional soccer players aiming to examine the effect of the independent variables (Caffeine vs. Placebo and Pre vs. Post plyometric and sled towing exercise) on CMJ performance on four time points: baseline, 1, 3 and 5 min. The CMJ was used as an indicator of neuromuscular performance in response to a series of plyometric exercise and sled towing which were used as conditioning activities. On the first visit, participants executed the conditioning activities and three CMJ as familiarization. In two subsequent visits they performed the same activities in two conditions: 60 min after the ingestion of a caffeine solution (5 mg.kg^− 1^) or placebo. For the CMJ, subjects were instructed to begin with hands on hips, squatting to a self-selected depth and immediately jump as high as possible. Two attempts were given with 15 s of rest in between. The average performance of the 2 jumps was used for statistical analysis. Jump height was measured via flight time using a jump platform (Jump System, CEFISE, Brazil). Fifteen seconds recovery was given between each CMJ. Prior to beginning the study, CMJ jump performance data was collected with all club players and a high intraclass correlation coefficient for jump height was found (*r* = 0.921, two-way mixed model used). The protocol used in this study was similar to those used in previous investigations of CMJ performance [19, 36].

### Design and procedures

All volunteers were asked to abstain from foods and liquids containing caffeine, as well as any nutritional supplements for at least 24 h prior to test sessions. During the duration of the study, all athletes were living at the club facility during weekdays, where each volunteer followed their dietary prescription for breakfast prior to testing sessions, which were replicated at testing sessions. Also, caffeine absence was assured as all food were provided by the club staff. All testing took place between 10 am and 12 pm in the morning to control for any diurnal variation. Before testing, volunteers performed a generic warm-up consisting of 10 bodyweight squats, 10 forward lunges each side, 3 min of dynamic stretching of relevant lower limb musculature, and 5 submaximal CMJs. 1 min after warm-up, two baseline pre-conditioning activity exercise CMJs were recorded.

One hour before testing, participants ingested 5 mg kg^− 1^ of caffeine (Sigma-Aldrich, Sydney, USA) diluted in 250 mL of artificially sweetened water or a placebo drink consisting of 250 mL of artificially sweetened water. It was demonstrated that blood caffeine levels reach its peak approximately 60 min after ingestion [15]. The conditioning stimulus consisted of 2 sets of 15 ankle hops, 3 sets of 5 hurdle hops, and 3 sets of 20-m sprints with sled towing resulting in a total of 45 jumps and 60 m sprinting with external load. Ankle hops were performed with a stiff leg action and a fast reactive rebound off the floor bilaterally. Hurdle hops involve a tuck jump movement to clear the height of each hurdle, set at 50 cm, performed as fast as possible. For the sled towing a load of 15% of body weight was used, which is considered a moderate load [28] and is commonly used by these athletes in their training sessions. Thirty seconds rest was given between each set of every activity. These exercises were selected as they reflect the typical exercises performed by these players in their training program.

CMJ height was recorded as the dependent variable and was used in the analysis to compare the post-intervention performance after placebo or caffeine ingestion. Previous investigations demonstrated that plyometric exercises enhance CMJ performance in a 5 min window [6, 36]. As the objective of the current study was to investigate if caffeine ingestion promotes an additional effect, we choose 4 time points: baseline, 1, 3 and 5 min post conditioning activity.

### Statistical analyses

Two-way repeated measures analysis of variance (two-way ANOVA) was used to assess differences in CMJ performance post caffeine and placebo ingestion at four moments: before the PAP protocol and 1, 3 and 5 min post-PAP. Bonferroni post-hoc multiple comparisons were used and partial eta squared (η_p_^2^) was used as a measure of effect size. Alpha level was set at *P* = 0.05 a priori and SPSS version 21 (SPSS Inc., Chicago, USA) was used for analyses.

Magnitude-based inferences [4] were used to determine differences between baseline and T1, T3, and T5 moments. Inferences were calculated using the differences on mean values and the 90% confidence intervals. A change of 1 cm at jump height was considered the smallest worthwhile change (positive: + 1 cm; or negative: − 1 cm).

## Results

**Fig. 1:**
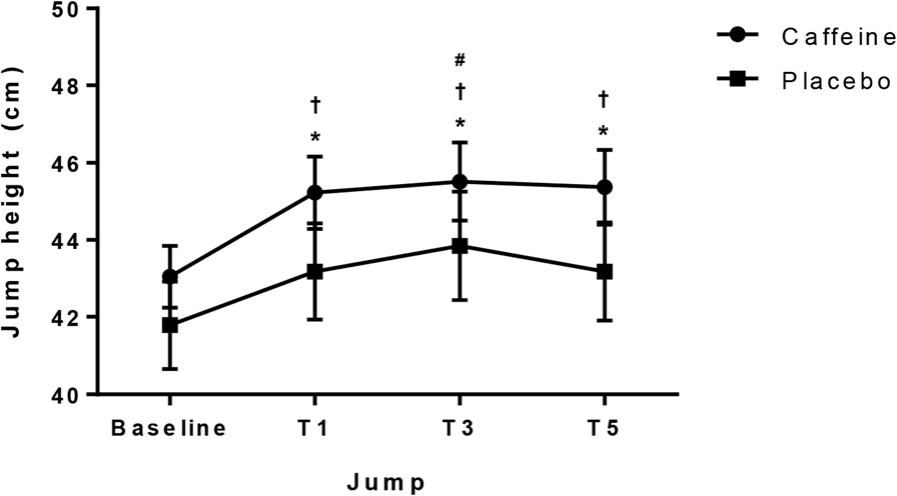
Coutermovement jump performance in caffeine and placebo condition. *P < 0.05 caffeine vs placebo; †*P* < 0.05 from baseline in caffeine condition; #*P* < 0.05 from baseline in placebo condition

Repeated measures ANOVA indicated a significant difference in CMJ height after the PAP protocol (F[3, 33] = 14.99, *P* < 0.001, partial η^2^ = 0.577). Analysis also indicated a significant difference on CMJ height across conditions, with caffeine eliciting a greater response (F[1, 11] = 10.12, *P* = 0.009, partial η^2^ = 0.479). No interaction effect was found (F[3,33] = 0.52, *P* = 0.667, partial η^2^ = 0.045). Bonferroni post-hoc comparisons indicated that the protocol increased jump height 1, 3 and 5 min after the conditioning activity in caffeine condition (T1: 5.07%, T3: 5.75% and T5: 5.40% compared to baseline; *P* < 0.05). In the placebo condition, jump height was significantly increased at T3 only (4.94% compared to baseline; *P* < 0.05) compared to baseline. Post-hoc analysis also indicated that jump height was higher in caffeine condition on T1, T3 and T5 compared to placebo (*P* < 0.05), but not on baseline (*P* > 0.05). These results are presented in Fig. 1.

Magnitude-based inferences demonstrated that the PAP protocol seems to be more effective in caffeine condition. In placebo condition, analysis indicated a probability of being positive of 59.3, 72 and 59.2% in T1, T3 and T5, respectively, compared to baseline. In caffeine condition, the probability of being positive were 82.6, 86.7 and 84.8% in T1, T3 and T5, respectively, compared to baseline (Table 2). When caffeine and placebo conditions were compared at baseline and 1, 3 and 5 min post potentiating stimulus, the probability of caffeine ingestion being positive at CMJ performance were 57.3, 74.6, 64.8 and 76.7%, respectively (Table 3).

**Table 2:** Magnitude-based inference analysis of results from countermovement jump performance in caffeine and placebo conditions at 1, 3 and 5 min post potentiating stimulus

	Probability of being positive, %	Probability of being trivial, %	Probability of being negative, %
Placebo			
Baseline to 1 min after	59.3	32.3	8.4
Baseline to 3 min after	72	22.8	5.2
Baseline to 5 min after	59.2	32.2	8.6
Caffeine			
Baseline to 1 min after	82.6	16.5	0.9
Baseline to 3 min after	86.7	12.6	0.7
Baseline to 5 min after	84.8	14.4	0.8

**Table 3:** Magnitude-based inference analysis of caffeine vs placebo comparison in countermovement jump performance at baseline, and 1, 3 and 5 min post potentiating stimulus

	Chance to be favorable at caffeine condition		
	Probability of being positive, %	Probability of being trivial, %	Probability of being negative, %
Baseline Caffeine vs placebo	57.3	36.7	6.0
1 min after Caffeine vs placebo	74.6	22.2	3.2
3 min after Caffeine vs placebo	64.8	28.2	7.0
5 min after Caffeine vs placebo	76.7	20.3	3.0

## Discussion

This study examined whether acute caffeine ingestion augmented the effect of a plyometric and sled towing potentiating stimulus on counter movement jump performance. This is the first study to examine this issue despite a theoretical basis for both caffeine and PAP to positively influence short-term explosive muscle performance. Results of this study indicate an improvement in CMJ height of 3.34, 4.94, and 3.34% at 1, 3, and 5 min after the conditioning activity without caffeine ingestion. Additionally, we demonstrated an improvement of 5.07, 5.75, and 5.40% after 1, 3, and 5 min when caffeine was ingested 60 prior to the conditioning activity. These results are novel and suggest that acute caffeine ingestion augments the effect of a preconditioning plyometric stimulus on CMJ performance in professional soccer players.

The magnitude of improvement after the conditioning activity in placebo condition was similar to that reported by Tobin and Delahunt [36]: 4.8, 3.9, and 3.5%, respectively, after plyometric exercises. De Villareal et al. [6] also reported a 6.96% enhancement of CMJ height 5 min after a volleyball-specific warm-up consisting of 25 repetitions of plyometric exercises. Heavy squat exercise also produces a PAP effect and promotes similar improvements on jump performance, with greater results seen in the parallel compared to the quarter squat [10]. To the best of our knowledge, the acute effect of sled towing on jump performance has not been investigated before. Herein we demonstrated that a combination of plyometric exercises and sled towing could improve CMJ performance in professional soccer players. Based on these observations it is clear that a variety of stimuli can contribute to the PAP effect, resulting in improved jump performance.

Data suggest that a minimum volume of exercise performed is necessary to induce a potentiation response. Wilson et al. [38] reported that in untrained individuals, a single set was more effective than multiple sets to induce improvements in power. On the other hand, in athletic populations, multiple sets seem to elicit a better response for a potentiation effect. Also, the potentiation effect after a conditioning activity appears to be dependent of the individual’s training status. Till and Cooke [34] evaluated the acute effects of plyometric exercises on CMJ performance and, contrary to our data, observed no enhancement. However, the conditioning activity consisted of only 5 tuck jumps, suggesting that the lower volume was not able to induce a potentiation effect.

Some have suggested that true muscle potentiation dissipates as quickly as 4 to 6 min after a conditioning exercise [22]. Similarly, a meta-analysis conducted by Wilson et al. [38] indicates that in general a rest interval of 7–10 min provides greater benefits than 3–7 min. However, when training status is considered, data shows that, in athletic populations, the greatest benefit is obtained after 3–7 min. This is different to non-athletic individuals, where the greatest PAP effect size is seen 7–10 min after the conditioning exercise [38]. In the present study, with trained athletes, CMJ performance was significantly increased 3 min post conditioning activity in the placebo condition. Conversely, in the caffeine trial, CMJ performance was increased 1, 3 and 5 min post activity and at these time points, performance was higher when compared to placebo condition. Moreover, magnitude-based inferences seem to support this claim, with higher probability of PAP protocol being positive to CMJ performance at caffeine condition, as well as of caffeine being positive to performance when compared to placebo. These data suggest that caffeine and PAP could have a synergistic effect on jump performance in trained individuals with moderate caffeine intake.

Previous research demonstrated that the ingestion of a caffeinated energy drink containing 3 mg caffeine.kg bw^− 1^ increased the mean jump height and muscle power during a 15-s jump test in semi-professional soccer players [7]. These results are in agreement with other studies using a diverse athletic population [1, 2, 8, 11, 13, 20, 27, 32]. At the present study, the 3% increase in CMJ height at caffeine trial compared to placebo was not significant (*P* > 0.05). However, during all other time points (T1, T3 and T5) this difference was statistically significant (4.74%, 3.80%, and 5.06%, respectively).

The mechanism underpinning the potentiation with heavy resistance exercise and plyometric stimulus appears to be the same [35]. The enhanced muscle performance after a conditioning exercise is thought to involve the phosphorylation of myosin regulatory light chain and the increase in recruitment of high-order motor units [29, 35]. It was also demonstrated that the mean pennation angle decreased 3 to 6 min after maximal voluntary contractions (from 16.2 to 14.4 degrees) [23]. Because a smaller pennation angle favors the force transmission for the tendons, acute modification on skeletal muscle architectural properties and the optimization of force transmission could also be involved in the phenomenon of PAP. Chronic adaptations to plyometric training involve the enhancement of muscular tension transmission and storage-recoil of elastic energy, reducing energy dissipation by the tendon [12]. However, additional research is needed to address the acute effects of a plyometric stimulus on the muscle-tendon unit and its contribution to enhanced muscle performance when used as a conditioning activity.

Several mechanisms have also been proposed for the caffeine-induced ergogenic effect during high intensity exercise were proposed involving peripheral and central mechanisms (for review see [5]). Earlier studies using incubated skeletal muscles demonstrated that milimolar concentrations of caffeine induce an ergogenic effect [21] with greater mobilization of calcium from sarcoplasmic reticulum [30]. This concentration, however, is supraphysiological and toxic in humans and it is unlikely that a greater mobilization of calcium is responsible for the caffeine-induced enhance muscle contractility. In humans, blood caffeine concentration rise to 10–70 uM, after the ingestion of 3 to 9 mg.kg bw^− 1^ [14]. More recent data supports the notion that micromolar concentrations of caffeine can elicit a small but significant increase in power output (3 to 6%) in isolated skeletal muscles [33]. However, the mechanism by which milimolar concentrations of caffeine enhance skeletal muscle contractility in vitro is still not clear.

Albeit local mechanisms have been proposed to enhance skeletal muscle contractility in response to caffeine, a favorable hypothesis involves the stimulatory effect of caffeine on the central nervous system (CNS) through the antagonism on adenosine receptors, particularly A1 and A2a receptors [17]. This blockade could result at the withdrawal of the adenosine effects on CNS, resulting in the stimulation of alertness and arousal and reducing pain perception at the presence of caffeine [3]. Based on the aforementioned mechanisms, based on current knowledge it is not possible to address how caffeine can enhance PAP response. It was postulated that after a conditioning stimulus, fatigue and potentiation can coexist and the net balance between them can have influence on the performance outcome [29]. At our understanding, two scenarios are possible: caffeine could stimulate skeletal muscle contractility and/or attenuated fatigue during high-intensity muscle contractions.

We have demonstrated here in a synergistic effect of PAP and caffeine ingestion on CMJ performance. However, we did not investigate the mechanisms involved in this effect. Based on previous research, it’s possible that PAP and caffeine ingestion promote increase in muscle contractility through distinct mechanisms, which can include those mentioned above. Although the present study identifies that the PAP response is augmented in the presence of caffeine, future research is needed which explicitly examines the underlying mechanism for the synergistic effect of caffeine ingestion and PAP observed in the present study. Some limitations of the present study include no investigation on the mechanisms behind the synergistic effects of PAP and caffeine ingestion, and data regarding blood caffeine levels in response to placebo and caffeine ingestion. However, the effects of moderate doses of caffeine on plasma concentrations are well documented in the literature [15].

## Conclusion

The addition of strength and/or plyometric exercises in warm-up routines for sport events that depend on strength and power is widely spread. From a practical point of view, the PAP should not provide an enhanced performance during the entire soccer game (or other team sports) since the potentiation effect seems to dissipate quickly. However, if added to the warm up routine, PAP might benefit athlete’s performance during the initial stages of the game. Importantly, PAP has been suggested as a half-time strategy to improve performance during the initial stages of the second-half [31], a research has demonstrated that important aspects of performance are affected especially during the first 15 min of second-half [25, 37]. Caffeine has been used as an ergogenic to enhance strength and power [5], and the dose used in this study (5 mg.kg^− 1^) has proven safe [26]. Although individual athlete responses should be considered, the synergistic effects of PAP and caffeine ingestion may be beneficial for athletes enrolled in activities that depend on strength and power. The combination of caffeine ingestion alongside a potentiating conditioning stimulus also offers strength and conditioning coaches a practical and legal means by which short-term, explosive exercise performance may be enhanced for up to 5 min. A warning should be given to athletes from the National Collegiate Athletic Association (NCAA) regarding the use of caffeine, as it is considered a controlled substance for their athletes.

## Abbreviations

CMJ: Countermovement jump
PAP: Post-activation potentiation
